# Small Papillae Regulated by ***SPD25*** are Critical for Balancing Photosynthetic CO_2_ Assimilation and Water Loss in Rice

**DOI:** 10.1186/s12284-023-00676-7

**Published:** 2023-12-13

**Authors:** Lin Zhu, Faliang Zeng, Yinpei Liang, Qi Wang, Hongwei Chen, Pulin Feng, Mingqian Fan, Yanshuang Cheng, Jiayu Wang

**Affiliations:** Key Laboratory of Rice Biology & Genetic Breeding in Northeast China (Ministry of Agriculture and Rural Areas), Rice Research Institute of Shenyang Agricultural University, Shenyang, 110866 P. R. China

**Keywords:** Small papillae, Chlorophyll fluorescence, Water loss, Gas exchange, Optical properties, Rice

## Abstract

**Background:**

The leaf epidermis plays an important role in the transmission of light and the regulation of water and gas exchange, which influences the photosynthesis of mesophyll cells. Small papillae (SP) are one of the important structural elements of the leaf epidermis. The mechanism of the effect that small papillae have on rice leaf photosynthetic performance remains unclear.

**Results:**

In this study, a *small papilla deficient 25* (*spd25*) mutant was isolated from *japonica* rice Longjin1. Small papillae were absent on the adaxial and abaxial leaf surfaces of the *spd25* mutant and the silicon and cuticular wax content in the *spd25* mutant leaves decreased. Map-based cloning and functional analysis revealed that *SPD25*, encoding a guanine nucleotide exchange factor for Rop, is a novel allele of *OsRopGEF10*. The *spd25* mutant showed an increased water loss rate and reduced relative water content. The lower stomatal conductance in the *spd25* mutant prevented water loss but decreased the intercellular CO_2_ concentration and net assimilation rate. The fluorescence parameters showed that the inhibited CO_2_ assimilation reaction feedback regulated the photochemical electron-transfer reaction, but the performance of Photosystem II was stable. Further analysis indicated that the excess light energy absorbed by the *spd25* mutant was dissipated in the form of non-photochemical quenching to avoid photodamage through the optical properties of small papillae.

**Conclusions:**

*SPD25* regulates the development of small papillae on the surface of rice leaves, which play an important role in balancing photosynthetic gas exchange and water loss. This study deepens our understanding of the physiological mechanisms by which small papillae affect photosynthetic performance.

**Supplementary Information:**

The online version contains supplementary material available at 10.1186/s12284-023-00676-7.

## Background

As the global population grows, sustainable production of food on limited arable land is one of the challenges we face (Tilman et al. 2011). Plants use energy from photons to produce biochemical energy and to store carbon-based molecules as a source of chemical energy. In rice, the shape, size and epidermal structure of leaves, which serve as the main organ of photosynthesis, directly affect the growth and yield of the rice. The multiple specialized cells and structures of the leaf epidermis can resist biotic and abiotic stress. At the same time, the regulation of light reflection and water and gas exchange by epidermal structures also affects the photosynthetic performance of plants (Karabourniotis et al. 2021). For example, trichomes can intercept strong light and ultraviolet radiation, but also reduce light energy absorption and photosynthetic electron transfer rate (Yan et al. 2012; Ehleringer and Bjorkman 1978). The increase in stomatal opening in the epidermis can provide sufficient CO_2_ for photosynthesis and also lead to water loss (Gardner et al. 2023; Rao et al. 2015). Therefore, to optimize the photosynthetic performance of plants, the study of leaf epidermal structure still needs to continue.

Cuticular papillae (CP), dense exosomes formed on the cell wall of the leaf epidermis, are important structures of the leaf epidermis (Paradiso et al. 2011; Chowdhury et al. 2014). Assaad et al. (2004) found that the *PEN1* was actively transported to papillae to prevent fungal infection in the experiment of fungal infection of Arabidopsis thaliana, and the formation of papillae of the *pen1-1* mutant was delayed by 2 h, suggesting that *PEN1* has secretion and defense related functions and is necessary for the formation of papillae. The product encoded by the *Mildew Resistance Locus O* (*MLO*) gene in barley is a negative regulator of papilla formation, and papillae are considered to be a barrier against fungal invasion (Buschges et al. 1997; Huckelhoven et al. 1999; Maekawa et al. 2014). At an early stage of leaf development in the rice *bgl* mutant, OsRopGEF10 activates Rop/Rac GTPases to turn on the molecular signaling pathway for small papillae development (Yoo et al. 2011). In addition to acting as versatile signaling switches, the plant-specific Rho of plant (Rop) subfamily of Rho GTPases also plays important roles in signaling in an array of cellular processes including cell polarity establishment, intracellular trafficking, pollen tube and root hair growth, actin dynamics, H_2_O_2_ production, and hormone responses (Zheng and Yang 2000; Yang 2002; Kim et al. 2020; Liu et al. 2023). The molecular mechanism of papilla development has been widely studied, but functional research on papillae has mostly focused on defense against pests and diseases. As one of the epidermal structures in direct contact with the outside world, its optical properties and physiological function of regulating water and gas exchange have not been researched in depth.

It is worth noting that in the *bgl* mutant of rice, the leaf epidermis reflects more green and red light due to the absence of small papillae (Yoo et al. 2011). The photon absorption of chloroplasts depends not only on the distribution of photosynthetic pigment concentration but also on its absorption spectrum (Kume 2017). Green light may excite photosystems in the deep cell layer, making the light distribution of leaves more uniform, which is conducive to photosynthesis in leaves (Nishio 2000). In contrast, blue and red photons are less efficient and more likely to dissipate in the form of heat. Although heat dissipation is the most effective first line of defense against photodamage, it also leads to a significant loss of light energy utilization efficiency (Saccon et al. 2020). It is a reliable method to study the utilization of light energy capture in leaves by analyzing the dynamic curve of fluorescence rise through JIP test theory (Li et al. 2005). The photosynthetic electron-transfer reaction and carbon assimilation reaction in photosynthesis are closely related and affect each other (Petroutsos et al. 2016). The higher silicon content in CP plays an important role in controlling water loss (Wang et al. 2021). Therefore, the effect of small papillae on plant photosynthetic performance should be comprehensively considered from the aspects of light absorption, energy distribution and dissipation, and regulation of water and gas exchange.

In the present study, a rice mutant *spd25* with a deficiency in small papillae was isolated. This mutant was obtained by ethyl methane sulfonate (EMS) mutagenesis in *japonica* rice Longjin1 (Wild-Type, WT). The mutation of *SPD25* led to the absence of small papillae in the leaf epidermis, affecting the deposition of silicon and epidermal wax. To clarify the effect of SP on the photosynthetic performance of the *spd25* mutant, the physiological characteristics of SP in terms of light energy conversion, water loss, and gas exchange were further explored. This study provides a helpful basis for studying the physiological mechanism of the effect of small papillae on the photosynthetic performance of leaves.

## Results

### Phenotypic Characteristics and Main Agronomic Traits of the *spd25* Mutant

**Fig. 1:**
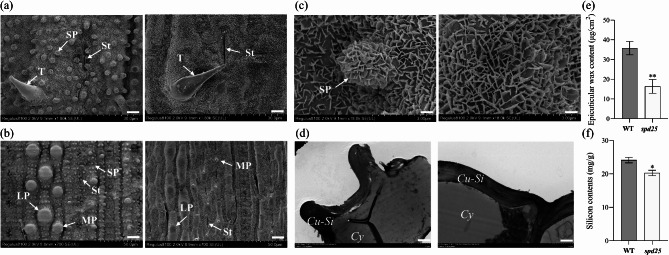
Phenotypes of WT and *spd25* mutant. **(a)** SEM images of the adaxial leaf surfaces in WT (left) and *spd25* (right). T, trichome; St, stomata; SP, small papillae. Bars = 60 μm. **(b)** SEM images of the abaxial leaf surfaces in WT (left) and *spd25* (right). MP, medium papillae; LP, large papillae. Bars = 150 μm. **(c)** Magnification of SP in (a). Bars = 6 μm. **(d)** TEM images of the epidermal cell wall in WT (left) and *spd25* (right). *Cu–Si*, cuticle-silica double layer; Cy, cytosol. Bars = 10 μm. **(e)** Epicuticular wax and **(f)** silicon contents in WT and *spd25* leaves. Shown are the mean ± SD from three biological replicates each containing ten plants. * and ** indicate *P* < 0.05 and *P* < 0.01, Student’s t-test

The *spd25* mutant was successfully obtained by EMS mutagenesis of Longjin1(WT). In brief, the bright green leaves were used as the index for screening *spd25* in fields because the leaves without SP would reflect more green light to make the leaves bright green (Yoo et al. 2011) (Figure S1a). CP can be classified by size as small (SP; 1.5–4.4 μm), medium (MP; 9–18 μm), and large papillae in rice leaves (LP; 21–30 μm) (Zhang et al. 2012). Scanning electron microscopy (SEM) images showed that SP developed on the adaxial and abaxial surfaces of WT leaves and was covered with waxy crystals (Fig. 1a-c). In contrast, MP and LP only existed on the abaxial leaf epidermis and were exposed (Fig. 1b). SP were absent on the adaxial and abaxial leaf surfaces of the *spd25* mutant compared with the WT, but other structures were normal (Fig. 1a, b). The wax content of the *spd25* mutant was significantly reduced by 54.36% compared with that of the WT (Fig. 1e). On the other hand, the transmission electron microscopy (TEM) images showed that SP were formed by the outward growth of the cuticle-Si double layer in the WT leaves (Fig. 1d). The silicon content of the *spd25* mutant was significantly lower than that of WT by 15.95% (Fig. 1f).

There were no significant differences in heading date, tiller number, panicle length or number of grains per panicle between the *spd25* mutant and WT (Figure S1b-e). However, the seed setting rate, 1000-grain weight and grain yield per hole of the *spd25* mutant were significantly 13.16%, 13.54% and 25.04% lower than those of the WT, respectively (Figure S1f-h).

### Genetic Analysis, Map-Based Cloning and Functional Verification of *SPD25*

**Fig. 2:**
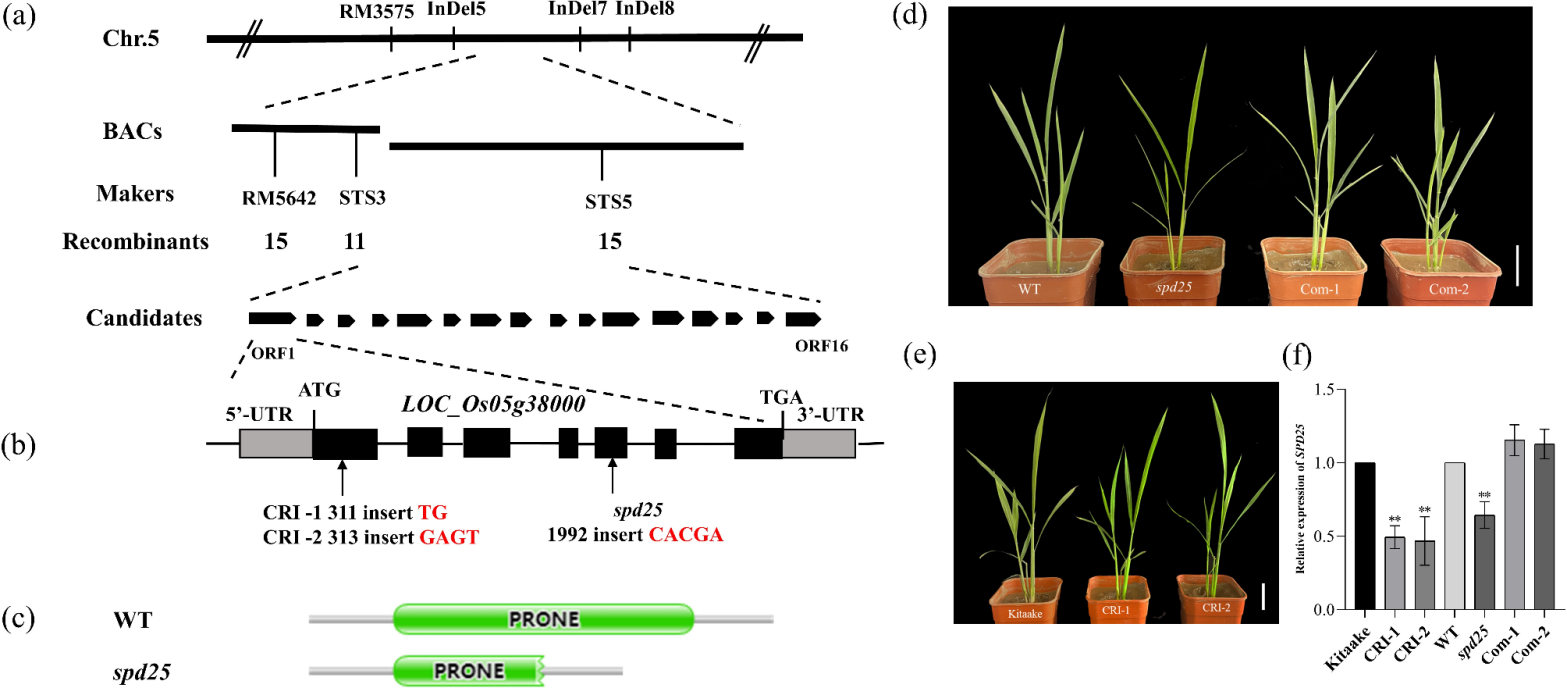
Map-based cloning of *SPD25*. **(a)** Fine mapping of *SPD25*. **(b)** Structure of the candidate gene *LOC_Os05g38000*. The red arrow represents the mutation site of *SPD25* in *spd25*, CRI-1 and CRI-2. **(c)** Structures of the SPD25 protein in WT and *spd25*. The SPD25 protein contains a PRONE domain. **(d)** Phenotype of WT, *spd25* and complementation lines at early tillering stage. Com, Complementation. Bars = 5 cm. **(e)** Phenotype of CRISPR/Cas9 transgenic lines at early tillering stage. CRI, CRISPR/Cas9. Bars = 5 cm. **(f)** Relative expression level of *SPD25*. Shown are mean ± SD from three biological replicates each containing ten plants. * and ** indicate *P* < 0.05 and *P* < 0.01, Student’s t-test

We constructed reciprocal crosses between the *spd25* mutant and Habataki and performed genetic analysis. The papillae on the leaf epidermis of all F_1_ plants developed normally. In the F_2_ population, the ratio of normal plants to small papillae deficient plants was 3:1 (Table S1). These results indicated that the *spd25* phenotype is controlled by a single recessive nuclear gene. Using 365 F_2_ mutant individuals, the *SPD25* locus was mapped to a 90.87-kb region on chromosome 5 between SSR markers STS3 and STS5 (Fig. 2a). Sequencing of 16 candidate genes in this region revealed a 5 bp nucleotide insertion mutation in exon 5 of *LOC_Os05g38000*, which resulted in a frameshift mutation and early termination of transcription (Fig. 2b). By searching the information on the *Oryza sativa japonica* group, it was found that the gene corresponds with *LOC_Os05g38000*, identified as *OsRopGEF10*. *SPD25* is a novel allele of *OsRopGEF10*.

To verify that the mutation in the *SPD25* gene induces the papilla-deficient phenotype of the *spd25* mutant, we constructed a complementation vector containing the entire genomic sequence of *SPD25* and transferred it into the *spd25* mutant by *Agrobacterium*-mediated transformation. All 20 independent transgenic plants obtained showed a wild-type phenotype (Fig. 2d, S2c, g). Furthermore, we knocked out *SPD25* in conventional varieties by using the CRISPR/Cas9 system. We obtained two independent transgenic lines that all carried homozygous mutants, including 2-bp and 4-bp insertions in exon 1 (Fig. 2b). These lines presented a phenotype similar to that of *spd25*, and neither the adaxial nor abaxial small papillae of the leaves developed (Fig. 2e, S2d, h). The expression level of *SPD25* in CRI-1 and CRI-2 was significantly reduced compared with Kitaake, whereas the expression in the Com-1 and Com-2 complementation lines returned to the level in the WT (Fig. 2f). Therefore, *LOC_Os05g38000* was confirmed to be the *SPD25* gene.

### Phylogenetic Analysis of SPD25

**Fig. 3:**
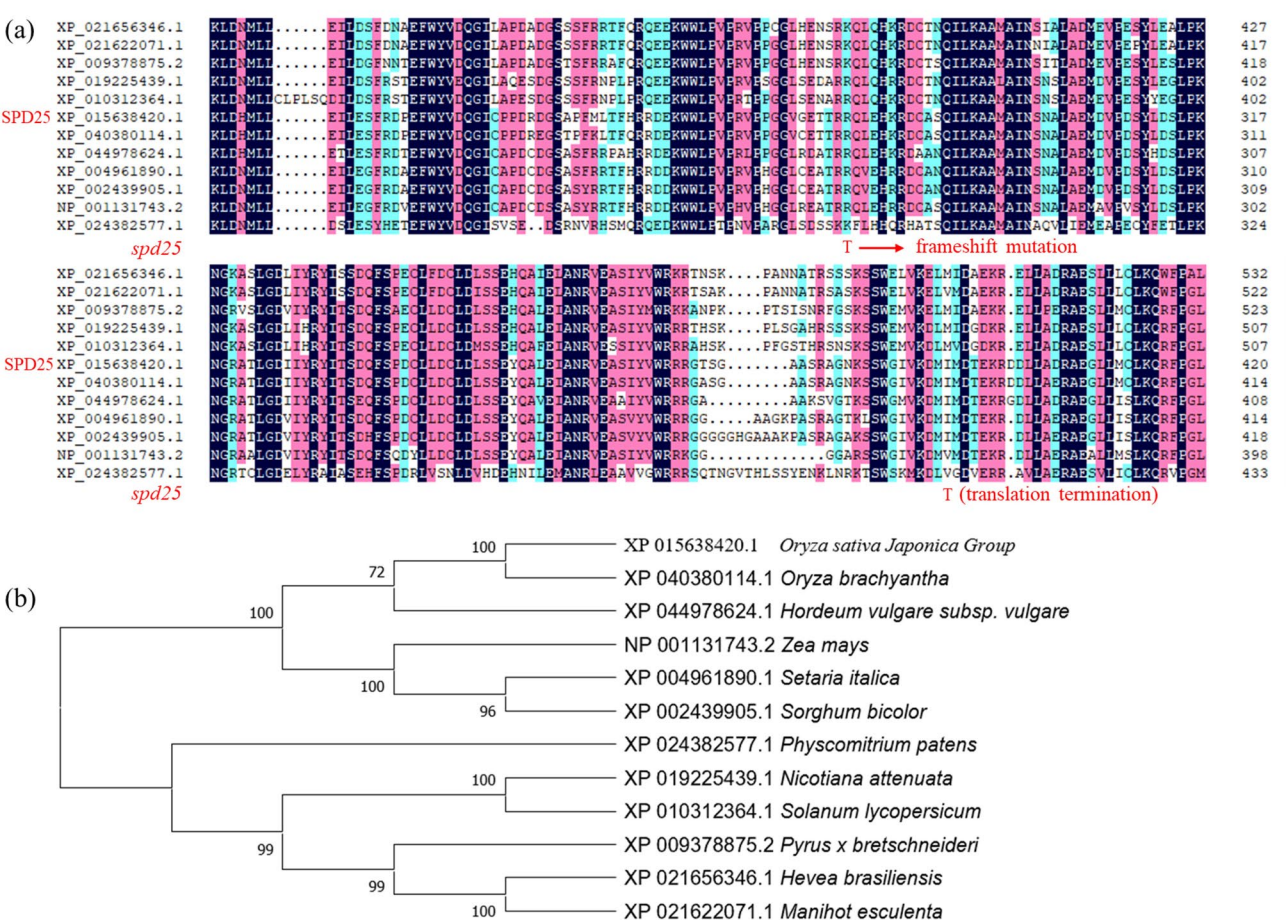
Phylogenic analysis of SPD25. **(a)** Amino acid sequence alignment of 12 types of SPD25 homologs. Protein sequences are *Hevea brasiliensis* (XP_021656346.1), *Manihot esculenta* (XP_021622071.1), *Pyrus x bretschneideri* (XP_009378875.2), *Nicotiana attenuata* (XP_019225439.1), *Solanum lycopersicum* (XP_010312364.1), *Oryza sativa* (XP_015638420.1), *Oryza brachyantha* (XP_040380114.1), *Hordeum vulgare subsp. Vulgare* (XP_044978624.1), *Setaria italica* (XP_004961890.1), *Sorghum bicolor* (XP_002439905.1), *Zea mays* (NP_001131743.2), *Physcomitrium patens* (XP_024382577.1). (The red arrow indicates the amino acid residue mutated in the *spd25* mutant). **(b)** Phylogenic tree of SPD25 and its homologs

Protein domain predictions using Pfam(https://pfam-legacy.xfam.org/) showed that SPD25 contained a conserved domain named PRONE (plant-specific Rop nucleotide exchanger) (Fig. 2c). BLAST-P analysis showed that SPD25 is highly conserved in plants including *Oryza brachyantha, Hordeum vulgare subsp. Vulgare, Zea mays, Setaria italica, Sorghum bicolor, Physcomitrium patens, Nicotiana attenuata, Solanum lycopersicum, Pyrus x bretschneideri, Hevea brasiliensis* and *Manihot esculenta* (Fig. 3a). Phylogenetic analysis of SPD25 was performed to investigate the evolutionary relationship among SPD25 homologs. As shown in Fig. 3b, SPD25 is closely related to both monocot and dicot homologs, suggesting that SPD25 is highly conserved in plants.

### Analysis of Chloroplast Development and Photosynthetic Pigments in the *spd25* Mutant

**Fig. 4:**
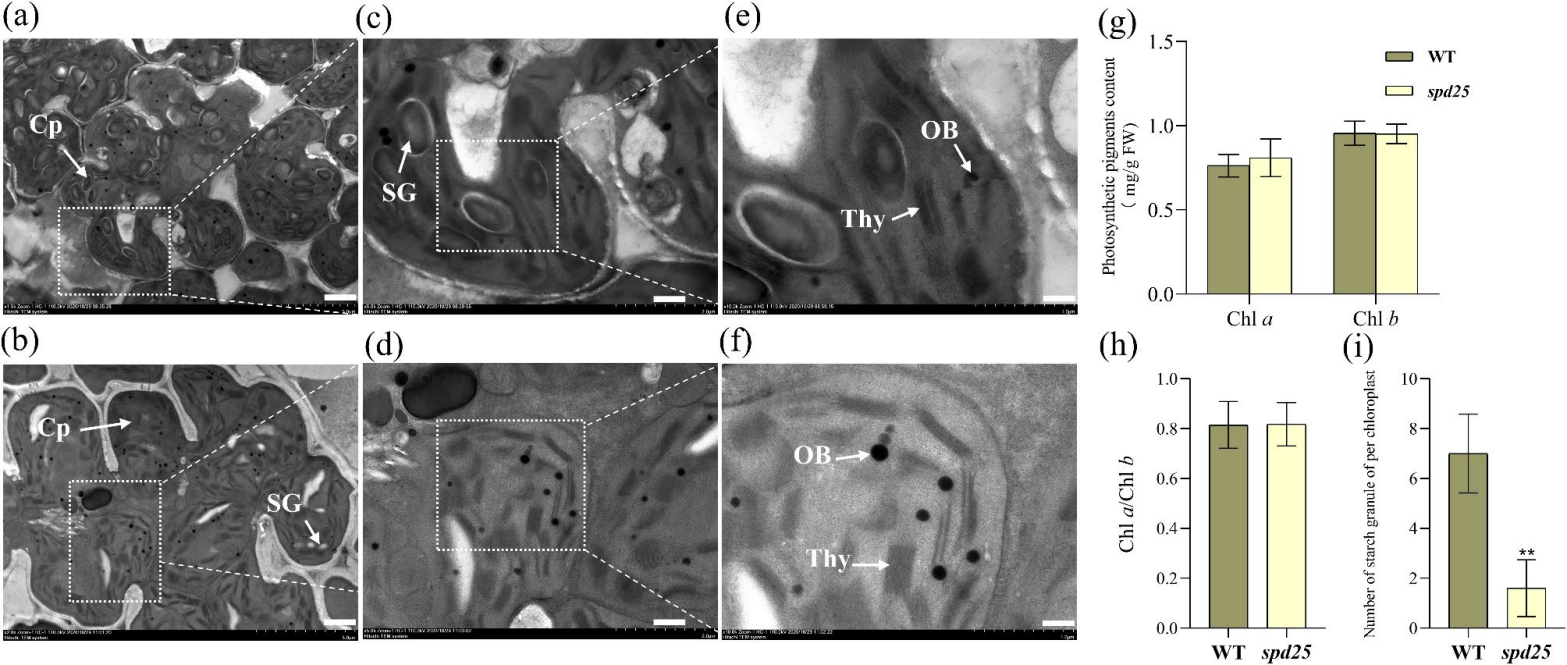
Analysis of chloroplast development and photosynthetic pigments content in the WT and *spd25*. (a − f) TEM images of the chloroplast in WT (**a**, **c**, and **e**) and *spd25* (**b**, **d**, and **f**). Cp, chloroplast; SG, starch granule; Thy, thylakoid lamellae; OB, osmophilic body. Bars (**a**, **b**) 20 μm; (**c**, **d**) 6 μm; (**e**, **f**) 3 μm. (**g**, **h** and **i**) Photosynthetic pigments content, Chl *a*/Chl *b* and number of starch granule of per chloroplast in WT and *spd25*. Shown are the mean ± SD from three biological replicates each containing ten plants. * and ** indicate *P* < 0.05 and *P* < 0.01, Student’s t-test

The levels of chlorophyll *a* (Chl *a*), chlorophyll *b* (Chl *b*) and Chl *a*/Chl *b* in the *spd25* mutant were not significantly different from those in the WT (Fig. 4g, h). TEM images showed that the number and morphology of chloroplasts in the *spd25* mutant mesophyll cells were similar to those in the WT (Fig. 4a-f). Moreover, both the *spd25* mutant and WT have clear thylakoid lamellar structures and intact thylakoid membranes. These results indicated that the chloroplasts of the *spd25* mutant developed normally. However, there were significantly fewer starch granules in the *spd25* mutant than in the WT (Fig. 4i). This suggested that the synthesis or accumulation of organic matter in the *spd25* mutant was reduced.

### Analysis of Optical Properties of SP and Chlorophyll *a* Fluorescence Transient in the *spd25* Mutant

**Fig. 5:**
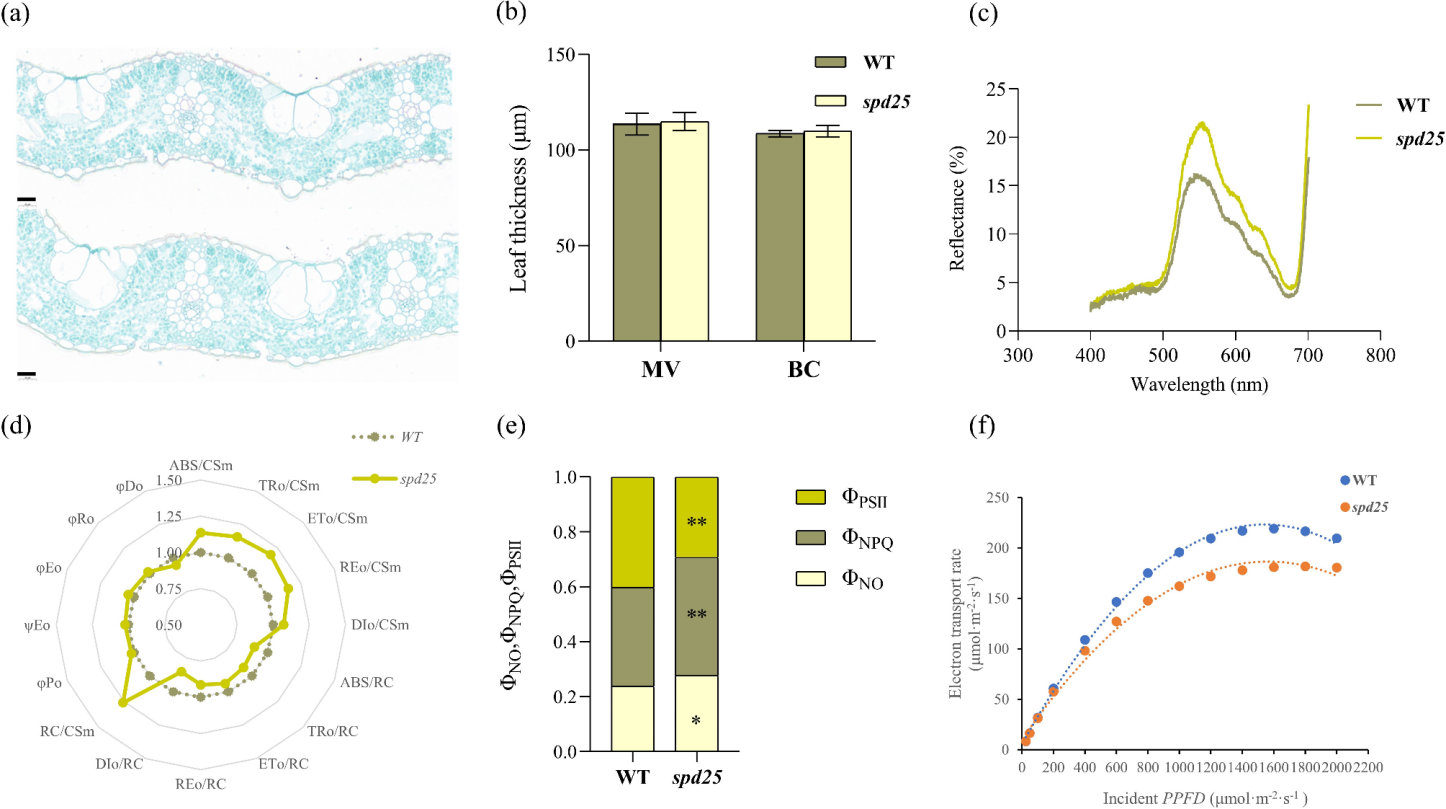
Analysis of slice images, reflectance and fluorescence parameter of WT and *spd25*. **(a)** Slice images of WT and *spd25* leaves. Bars = 20 μm. **(b)** Leaf thickness of WT and *spd25*. MV, minor vein region, BC, bulliform cell region. Shown are the mean ± SD from three biological replicates each containing ten plants. * and ** indicate *P* < 0.05 and *P* < 0.01, Student’s t-test. **(c)** Reflectance spectra of WT and *spd25* in visible light range (400–700 nm). **(d)** Relative changes of JIP-test parameters for WT and *spd25*. **(e)** Quantum yields of WT and *spd25.* Shown are the mean ± SD from three biological replicates each containing ten plants. * and ** indicate *P* < 0.05 and *P* < 0.01, Student’s t-test. **(f)** Light-response curves of photosynthetic electron transport rate for WT and *spd25*

Slice images showed that the thickness of the minor vein (MV) and bulliform cells (BC) in the leaves of the *spd25* mutant were almost the same as those of the WT (Fig. 5a, b). The reflectance of the *spd25* mutant was approximately 25–35% greater than that of the WT in the green light (500–600 nm) and red light (600–700 nm) regions (Fig. 5c).

To explore the effect of the optical properties of SP on light energy capture and utilization in leaves, the chlorophyll *a* fluorescence kinetics transient was analyzed by using the JIP-test theory. The absorption flux per reaction center (ABS/RC), trapped flux per reaction center (TRo/RC), electron transport flux per reaction center (ETo/RC) and dissipation energy flux per reaction center (DIo/RC) were significantly decreased by 11.01%, 9.06%, 5.84% and 17.07% in the *spd25* mutant compared with the WT, respectively (Fig. 5d, Table S2). However, the number of active reaction centers per cross-section (RC/CSm) in the *spd25* mutant significantly increased by 26.08% compared with that in the WT.

The absorption flux per cross section (ABS/CSm), trapped flux per cross section (TRo/CSm), electron transport flux per cross section (ETo/CSm), dissipation energy flux per cross section (DIo/CSm) and electron flux reducing end electron acceptors at the photosystem I (PSI) acceptor side per cross section (REo/CSm) were significantly increased by 13.55%, 15.71%, 18.42%, 7.29% and 15.47% in the *spd25* mutant compared to WT, respectively. The above fluxes per cross section were basically consistent with the increase in RC/CSm (Fig. 5d, Table S3).

There were no significant differences in the ratio of energy flux per absorption, such as the maximum quantum yield of primary photochemistry (φPo), efficiency with which a trapped exciton can move an electron into the electron transport chain further than QA (ψEo), quantum yield of electron transport (φEo), thermal dissipation quantum yield (φDo) and reduction of end electron acceptors of PSI (φRo) (Fig. 5d, Table S4). φPo reflects the maximal photochemical efficiency after dark adaptation, and its determination is consistent with that of the modulated fluorometer parameter *Fv*/*Fm*.

### Analysis of Energy Partitioning under Steady-State Photosynthesis in the *spd25* Mutant

The chlorophyll fluorescence parameters showed that there was no difference in *Fv/Fm* and *Fv ‘/Fm’* between the *spd25* mutant and WT in both dark and light adaptation states (Fig. 5d, Table 1). However, the fraction of opened PSII centers (*q*_P_) in the *spd25* mutant was significantly lower than that in the WT under the light-adapted state (Table 1). In the *spd25* mutant, 29% of the total energy was assigned to the quantum yields of PSII (Φ_PSII_), while in the WT, Φ_PSII_ was 40% of the total energy (Fig. 5f). In contrast, the other two components of the *spd25* mutant were significantly higher than those of WT, among which the quantum yields of non-photochemical quenching (Φ_NPQ_) increased more than the quantum yields of basal energy dissipation (Φ_NO_) (Fig. 5e). Table 2 shows that there was no significant difference in the initial slope (α_e_) of the response curve of electron transfer rate to incident PPFD between the WT and the *spd25* mutant. However, the maximum electron transport rate (ETR_max_) of the *spd25* mutant was 20.56% lower than that of the WT. When PPFD ≥ 1600, the ETR of *spd25* decreased more slowly than that of the WT (Fig. 5f).

**Table 1:** Chlorophyll fluorescence parameters at light intensity of 1000 µmol·m^− 2^· s^− 1^ in the flag leaves of WT and *spd25* at flowering stage. Shown are the mean ± SD from three biological replicates each containing ten plants. * and ** indicate *P* < 0.05 and *P* < 0.01, Student’s t-test

Materials	*Fv*’/*Fm*’	ΦPSII	*q*_P_
WT	0.60 ± 0.01	0.42 ± 0.04	0.65 ± 0.09
*spd25*	0.59 ± 0.02	0.30 ± 0.03 **	0.49 ± 0.07 **

**Table 2:** The fitted values of photosynthetic electron flow for WT and *spd25*. Shown are the mean ± SD from three biological replicates each containing ten plants. * and ** indicate *P* < 0.05 and *P* < 0.01, Student’s t-test

Parameters	WT	*spd25*
α_e_	0.3376 ± 0.0289	0.3436 ± 0.0229
ETRmax	219.2310 ± 19.7735	181.8420 ± 14.3319 *
PAR_sat_	1585.6800 ± 116.6944	1775.5400 ± 104.224
R-Squared	0.9981 ± 0.0032	0.9980 ± 0.0029

### Analysis of Leaf Water Content of the ***spd25*** Mutant

**Fig. 6:**
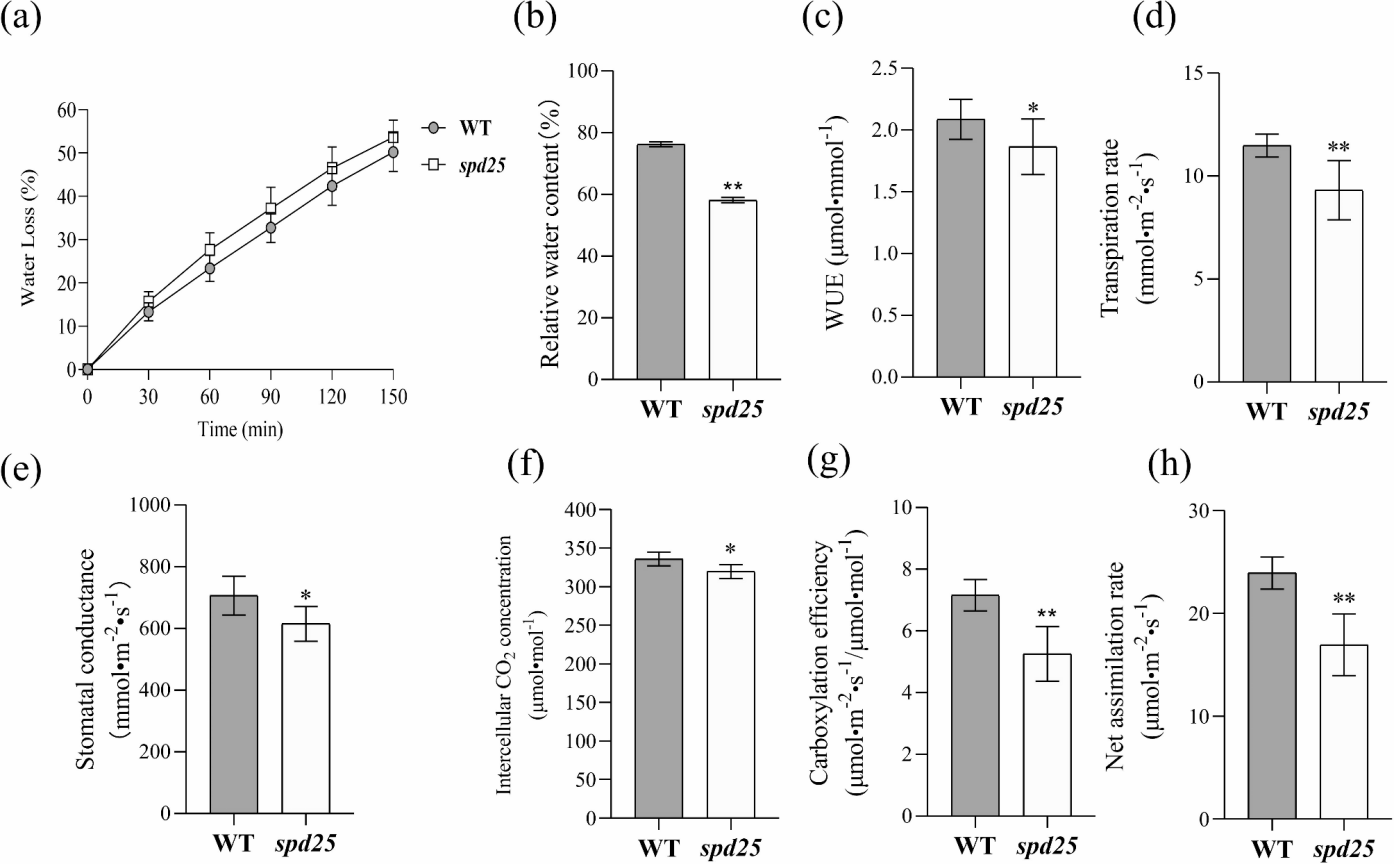
Analysis of water losses and gas exchange parameters in WT and *spd25*. **(a)** Contrast of water loss rate in leaves between WT and *spd25*. **(b)** Contrast of relative water content in leaves between WT and *spd25.* Shown are the mean ± SD from three biological replicates each containing ten plants. * and ** indicate *P* < 0.05 and *P* < 0.01, Student’s t-test. **(c)** Water use efficiency (WUE), **(d)** Transpiration rate (E), **(e)** Stomatal conductance (g_s_), **(f)** intercellular CO_2_ concentration (Ci), **(g)** carboxylation efficiency (CE) and **(h)** net assimilation rate (An) of WT and *spd25* at a PPFD of 1200 µmol•m^− 2^•s^− 1^. Shown are the mean ± SD from three biological replicates each containing ten plants. * and ** indicate *P* < 0.05 and *P* < 0.01, Student’s t-test

The rate of water loss from detached leaves was significantly increased in the *spd25* mutant (Fig. 6a). The relative water content of the *spd25* mutant leaves was significantly lower than that of the WT leaves (Fig. 6b). The leaf temperature of the *spd25* mutant was significantly higher than that of the WT (Figure S3).

### Analysis of CO_2_ Assimilation of the *spd25* Mutant

The water use efficiency (WUE), transpiration rate (E), stomatal conductance (g_s_), intercellular CO_2_ concentration (Ci), carboxylation efficiency (CE) and CO_2_ net assimilation rate (An) were significantly reduced by 10.58%, 15.05%, 12.89%, 3.3%, 21.12% and 23.65%, respectively, in the *spd25* mutant compared with the WT (Fig. 6c-h, Table S5).

## Discussion

### Dysfunction of *SPD25* Affects the Development of the Small Papillae on the Leaf Epidermis of *spd25*

The formation of specialized epidermal structures requires precise signaling guidance (Fornero et al. 2017). In this study, we cloned the *SMALL PAPILLAE DEFICIENT25*(*SPD25*) gene, which is a novel allele of *OsRopGEF10*. Multiple sequence alignment showed that the *spd25* mutation was within a conserved domain (Fig. 3a). SPD25 is closely related to both monocot and dicot homologs, suggesting that SPD25 is highly conserved in plants (Fig. 3b). The insertion of CACGA in the fifth exon of *SPD25* led to a frameshift mutation in the 277th amino acid and resulted in the premature translation termination of the encoded protein at the 392th amino acid of SPD25 (Fig. 3a).The product encoded by *SPD25* is the guanine nucleotide exchange factor OsRopGEF10, which activates Rop/Rac GTPases that function as molecular switches in signal transduction by replacing bound GDP with active GTP in eukaryotes in response to external or internal signals (Temple and Jones 2007). OsRopGEF10 can activate OsRac1, a small GTPase in the Rop/Rac family, to turn on the molecular signaling pathway for SP development (Yoo et al. 2011). This change resulted in the cleavage of the highly conserved PRONE domain of OsRopGEF10, which inhibited the function of OsRopGEF10 in the *spd25* mutant (Fig. 2c). The PRONE domain shows exclusive substrate specificity to members of the Rop family and has been shown to be necessary and sufficient to promote nucleotide release from Rop (Kikuchi et al. 2003). Although *SPD25* differed from *OsRopGEF10* in mutation type and site, both ultimately resulted in frameshift mutations and premature translation termination, disrupting the PRONE domain of OsRopGEF10. Therefore, mutation of *SPD25* may interrupt the signaling cascade of OsRopGEF10-OsRac1, resulting in the absence of SP on the leaf surface and affecting silicon deposition in the epidermal cell wall in the *spd25* mutant (Fig. 1a, b and f). Moreover, our study revealed that although the waxy structure of the leaf epidermis was normal (Fig. 1c), the wax content was significantly reduced in the *spd25* mutants (Fig. 1e).

### Effect of SP Deficiency on the Optical Properties and Light Energy Conversion of *spd25* Mutant Leaves

As one of the leaf epidermis structures, SP is the outermost boundary for receiving incident light. The optical properties of SP determine the reflectance, absorbance, and transmittance of the leaf, and thus the light intensity and spectral quality reaching photosynthetic tissues (Klancnik et al. 2014). Light intensity and spectral quality directly affect the energy conversion process of plant cells (Murata et al. 2007). In our study, JIP test parameters showed that under dark adaptation, the maximum quantum yield of primary photochemistry (φPo), the efficiency of excitation energy capture by PSII reaction centers (ψEo) and the quantum yield for electron transport (φEo) of the *spd25* mutant were almost the same as those of the WT (Fig. 5d). The increase in RC/CSm in the *spd25* mutant alleviates the burden of active reaction centers and significantly reduces specific energy fluxes (ABS/CSm, TRo/CSm, and ETo/CSm) (Kalaji et al. 2014). The TEM results showed that the chloroplasts of the *spd25* mutant were well developed and had normal photosynthetic structures (Fig. 4a-f). There were no significant differences in the Chl *a* and Chl *b* contents and Chl *a*/Chl *b* between the *spd25* mutant and WT, indicating that the size of the light-capture chlorophyll antenna of the *spd25* mutant and WT were similar. In the solar radiation spectrum, photosynthetically active radiation is typically defined as light with a wavelength range from 400 to 700 nm (McCree 1972). The *spd25* mutant leaf surface was smoother and reflected approximately 35% more green region (500–600 nm) and 25% more red region (600–700 nm) than the WT (Fig. 5c). Therefore, the transmission and absorption of green light and red light in the leaves of the *spd25* mutant were reduced. In summary, the increase in RC/CSm in the *spd25* mutant may be related to spectral reflection.

**Fig. 7:**
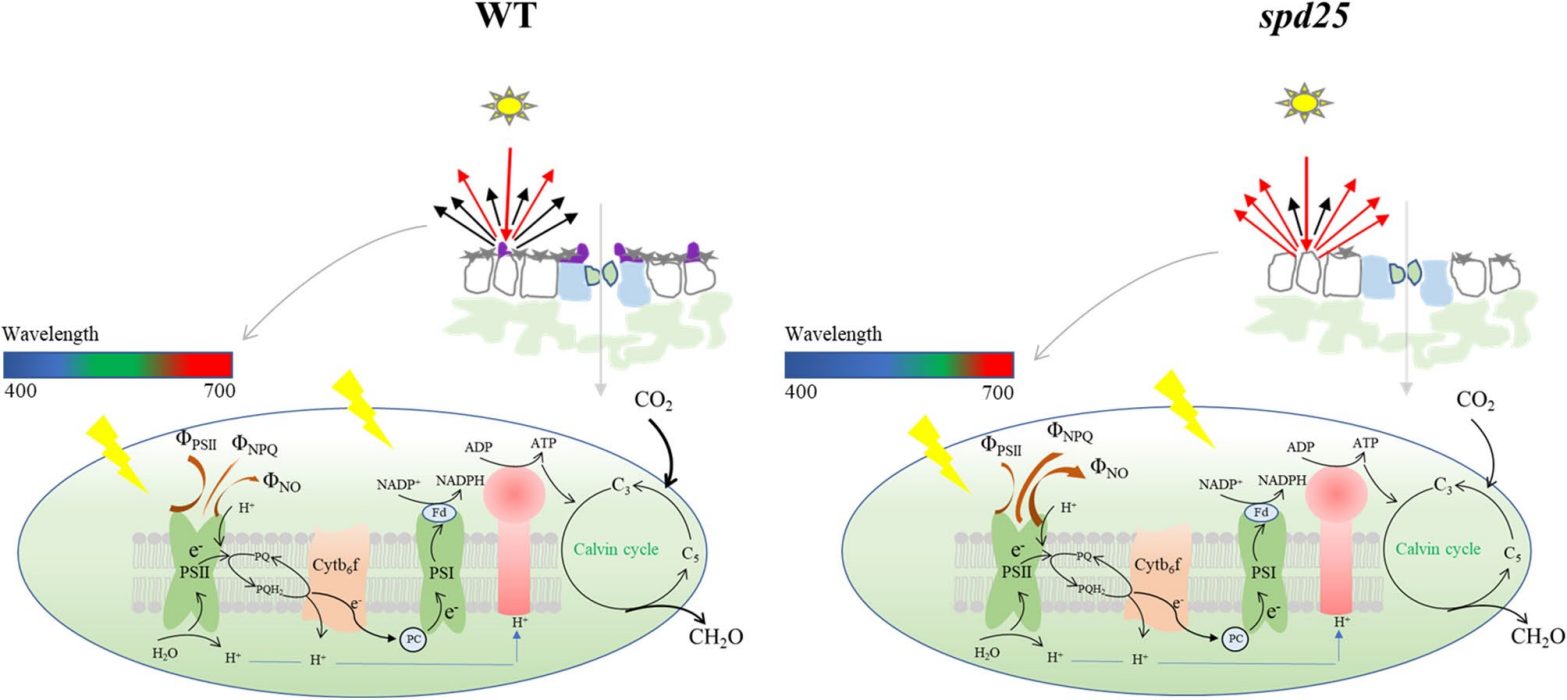
A model was proposed for physiological mechanism of the effect of small papillae on photosynthetic performance. The absence of SP in the *spd25* mutant affected silicon and epidermal wax deposition, thereby accelerating water loss in leaves, which lead to stomatal closure and reduced gas exchange parameters. The inhibited carbon assimilation reaction of photosynthesis in the *spd25* mutant feedback affected the primary reaction of photosynthesis. The optical properties of small papillae dissipate excess energy by up-regulating Φ_NPQ_ and Φ_NO_.

Under light-adapted states, *Fv ‘/Fm’* was not significantly different in the *spd25* mutant compared with the WT. *Fv/Fm* and *Fv ‘/Fm’* showed that the stability and maximum capacity of PSII in the *spd25* mutant were not affected before carbon assimilation activation (Kasajima et al. 2009; Lazar 2015). However, ΦPSII, *q*_P_ and ETR were significantly reduced in the *spd25* mutant compared with the WT. The photosynthetic electron-transfer reaction and carbon assimilation reaction in photosynthesis are closely related and affect each other (Petroutsos et al. 2016). Therefore, the feedback regulation of the carbon assimilation reaction to the light reaction may be the main reason for the inconsistent changes in fluorescence parameters under dark adaptation and light adaptation. Overexcitation of the photosynthetic system may result in photoinhibition or photodamage of the PSII reaction center (Adams III et al. 2002). The quenching analysis of PSII showed that under light adaptation, the energy allocated to the photochemical reaction in the energy absorbed by the PSII of the *spd25* mutant was significantly reduced, and the excess excitation energy was converted to thermal energy release (Fig. 5e). Although plants absorb less green light, green light can penetrate deeper into leaf tissues, thus giving it the potential to excite photosystems in deeper cell layers (Vogelmann and Evans 2002; Terashima et al. 2009). Under blue light, the paraxial excitation energy of lettuce leaves was found to be larger, leading to upregulation of NPQ (Liu and van Iersel 2021). The upregulation of Φ_NPQ_ in the *spd25* mutant dissipated excessive excitation energy and avoided photodamage to the photosystem, but it also wastes light energy. Therefore, we concluded that the inhibition of the photochemical reaction of the *spd25* mutant was mainly due to the feedback regulation of carbon assimilation, while the optical properties of SP played an important role in dissipating too much excitation energy and avoiding light damage (Fig. 7).

### Effect of SP Deficiency on Water Loss and Gas Exchange in Leaves of the *spd25* Mutant

The leaf surface plays a key role in protection against multiple stress factors such as water loss, overheating and insect or pathogen attack (Jian et al. 2022; Johnson et al. 2002). The cuticle-Si double layer formed by the leaf epidermis and cuticle can reduce transpiration loss in leaves (Hattori et al. 2005; Ma and Yamaji 2006; Wang et al. 2021). The water loss rate of the *spd25* mutant in isolated leaves increased significantly, and the relative water content decreased significantly, indicating that the *spd25* mutant had a poor water retention ability compared with the WT (Fig. 6a, b). The decrease in WUE indicated that the *spd25* mutant had poor drought tolerance and might face drought stress. It is well known that the cuticular transpiration of leaves will increase with the decrease in wax and silicon deposition in the epidermis (Gong et al. 2005). However, the transpiration rate of the *spd25* mutant was significantly lower than that of the WT. We speculated that the strong transpiration of the *spd25* mutant caused water loss faster than the rate of root water absorption from the soil, which led to stomatal closure to slow transpiration loss. We noticed that the leaf temperature of the *spd25* mutant was significantly higher than that of the WT (Figure S2). Leaves reduce surface temperature through transpiration, reduce respiratory consumption, and prevent damage to the photosynthetic system. The radiation absorption of a leaf arises as a consequence of absorption by chloroplasts (Hogewoning et al. 2012). High temperature weather and the release of photosynthetic radiation in the form of heat energy may aggravate transpiration and lead to water loss (Kume 2017). In addition, the absence of SP may reduce the wind resistance across the blade surface and accelerate transpiration (Maricle et al. 2009). To prevent leaf water loss, the stomatal opening of the *spd25* mutant was reduced or closed, which can be reflected by the reduced stomatal conductance (Roessler and Monson 1985).

Gas exchange parameters showed that the gs and Ci of the *spd25* mutant decreased while An decreased, indicating that the decrease in An was mainly affected by insufficient CO_2_ supply (Fig. 6e, f and h). If non-stomatal limitation factors play a dominant role, the intercellular CO_2_ concentration will increase with decreasing An and gs. The decrease in photosynthetic rate is an important factor affecting the yield of the *spd25* mutant. We also observed a significant decrease in carboxylation efficiency of the *spd25* mutant compared to WT. The carboxylation efficiency directly affects the assimilation of CO_2_ and is also related to water use efficiency, so further research is needed.

## Conclusions

A novel allele, *SPD25*, was cloned in rice, which regulates the development of small papillae on the leaf surface. The absence of SP in the *spd25* mutant affected the deposition of silicon and epidermal wax, resulting in a lower relative water content and higher water loss rate. However, the decrease in the transpiration rate in the *spd25* mutant indicated that stomatal opening was small or closed to prevent leaf water loss. Stomatal restriction was the main factor for the decrease in photosynthetic rate in the *spd25* mutant. The inhibited CO_2_ assimilation reaction feedback regulated the photochemical electron-transfer reaction, but the performance of photosystem II was stable. The excess light energy absorbed by the *spd25* mutant was dissipated in the form of non-photochemical quenching to avoid photodamage through the optical properties of small papillae. The findings of this study deepen our understanding of the physiological mechanism of the effect of small papillae on the photosynthetic performance of leaves.

## Materials and Methods

### Plant Materials and Growth Conditions

The small papillae deficient mutant 25 (*spd25*) was obtained from Longjin1 by ethyl methane sulfonate (EMS) inducible. The F_1_ population was derived from the cross between the *spd25* mutant and Habataki. The F_2_ population was obtained by F_1_ self-fertilization for fine mapping *SPD25* locus. In the F_2_ population, the plants with the mutant phenotype were selected for genetic mapping. The absence of small papillae made the leaves reflect more green light and appear bright green, which was more convenient for sampling in the field. All plants were grown in the field of the Rice Research Institute, Shenyang Agricultural University (41°N, 123°E). The experimental field was a sandy loam soil and irrigated with well water. Rice plants were cultivated in a 4.5 m × 3.6 m plot with a distance of 30 cm (row spacing) × 13.3 cm (hole spacing), and each sample was repeated three times. The total fertilization amount is 225 kg·ha^− 1^ urea, 120 kg·ha^− 1^ potassium sulfate and 220 kg·ha^− 1^ calcium phosphate. Management in the field followed normal standard agricultural practices.

### Transmission and Scanning Electron Microscopy

Fresh leaves from the WT and the *spd25* mutant at 30 days after sowing were cut into 1 mm × 2 mm strips and fixed in 2.5% glutaraldehyde with phosphate buffer (pH = 7.4) at 4 °C for 48 h under vacuum. These samples were washed with phosphate buffered and further fixed in 1% osmium tetroxide for two hours. After washing three times, the leaves were dehydrated with a gradient of ethanol. The dehydrated samples were soaked in SPI 812 embedding medium for ultrathin sections. The ultrathin sections were stained with 7.7% uranyl acetate and lead citrate for examining with a Hitachi HT7700 transmission electron microscope.

Fresh leaves were cut into 3 mm × 5 mm strips for the SEM. The steps of fixation and dehydration of the samples were the same as those described above. After dehydration, the samples were treated with 50%, 70%, 80% and 90% tert-Butanol once for 10 min each, and 100% tert-Butanol three times for 10 min each. The samples dried by freeze-drier (VFD-30) were coated with gold, and then were observed by a Hitachi Regulus 8100 scanning electron microscopy.

1 cm leaf in the middle of the flag leaf was collected and fixed in formalin acetic-alcohol solution. After gradient dehydration with ethanol, the samples were embedded in paraffin. The wax block was sliced and stained with 1% toluidine blue, and then scanned with digital full slide scanning. The scanned images were analyzed by using caseviewer software.

### Measurement of Chlorophyll, Silicon, and Epicuticular Wax Contents

0.5 g fresh leaves were cut and soaked in 15 ml of 95% alcohol and left in the dark for 48 h. The absorbance of 1mL samples was measured by spectrophotometer (HITACHI U-3900, Tokyo, Japan) at 663 nm and 645 nm, and repeated three times. The contents of chlorophyll *a* (Chl *a*) and chlorophyll *b* (Chl *b*) were calculated according to the methods described by Wellburn (Wellburn 1994).

Fresh leaves were fixed at 105℃ in the oven and dried at 70℃ for more than 3 days and ground into powder. 5mL 4 mol/L NaOH was distilled in nickel crucible with a graphite heating plate (DB-2EFS). The sample was weighed and spread 0.05 g on NaOH in nickel crucible, covered and placed on an electric heating plate for heating and ashing, then transferred to a high temperature electric furnace and melted for 10 min at 700–720 ℃. After cooling, it was transferred to a 50mL volumetric bottle containing 5mL 4 mol/L HCl for constant volume. The Si concentration was determined by the colorimetric molybdenum blue method at 600 nm (Tamai and Ma 2008).

2 g fresh leaves was weighed and the extraction of epicuticular wax on rice leaves was performed immediately after calculating their surface area. The samples were placed in 30mL chloroform at room-temperature and removed immediately after 30s. Then the treated samples were added to the same volume of 60℃ chloroform and removed after 30s. After the extract was volatilized naturally, the waxy mass was weighed. The ratio of wax mass to leaf surface area is the wax content.

### Genetic Analysis and Map-Based Cloning of *SPD25*

The F_1_ population was obtained by crossing the *spd25* mutant with Habataki for genetic analysis. A total of 365 mutant individuals were selected from F_2_ population to map the *SPD25* locus. Genomic DNA fragments of candidate genes were amplified from the *spd25* mutant and WT plants, sequenced and compared using MegAlign. These primer sequences are listed in Table S6.

### Total RNA Extraction and Quantitative Real Time PCR (qRT-PCR) Analysis

Total RNA was extracted from flag leaf using a Plant RNA Extraction Kit (TaKaRa, Dalian, China). The first-strand cDNA was synthesized using a Prime Script RT reagent kit (Takara Bio, Kyoto, Japan). The qRT-PCR was performed using a TB Green R Premix Ex TaqTM II (Tli RNaseH Plus) (TaKaRa, Dalian, China) in an Applied Biosystems QuantStudio 3 Real-Time PCR System (Thermo Fisher Scientific, United States). Three biological replicates were performed for each gene. The relative expression of each gene was analyzed using the 2^−ΔΔCt^ method. All primers sequences for qRT-PCR are listed in Table S6.

### Plasmid Construction and Plant Transformation

To produce the pCAMBIA1300-*SPD25* complementation constructs, the full-length coding sequences of *SPD25* were amplified and cloned into the pCAMBIA1300. The gene editing constructs of *SPD25* were produced by using the CRISPR/Cas9 system. *SPD25*-gRNA was cloned into the pRGEB32. The recombinant vector was transformed into the calli of Kitaake by Agrobacterium-mediated. All constructs used in this study were confirmed by sequencing. These constructions transformed by *agrobacterium tumefaciens*-mediated transformation of rice. The positive transgenic plants screened by hygromycin were sequenced to verify the mutation site. These primers are listed in Table S6.

### Phylogenetic Analysis

Homologues of *SPD25* were queried by blast analysis on the NCBI (https://www.ncbi.nlm.nih.gov). Multiple sequences were aligned, and a phylogenetic tree was constructed by MEGA6.0 software based on the neighbor-joining method (1000 replicates).

### Determination of Leaf Reflectance, Water Loss Rate in Vitro and Relative Water Content

Light reflectance of the leaves was measured using the spectrometer with a leaf clip (CI-710, United States). Spectral reflectance was analyzed in the visible light range (400–700 nm). The third leaf of rice growing for one and a half months was selected. The intact leaves were quickly immersed in ddH_2_O and weighed after sealed immersion for 6 h, that is, saturated fresh weight M1. Then the leaves were marked and placed in a dark environment, and weighed at 30, 60, 90, 120 and 150 min respectively, denoted as Mn. The water loss rate was calculated as:

1 $$\text{w}\text{a}\text{t}\text{e}\text{r}\,\text{l}\text{o}\text{s}\text{s}\,\text{r}\text{a}\text{t}\text{e}\,\left(\text{\%}\right)=\frac{M1-Mn}{M1}$$

The third leaf was selected as the material and weighed as M1. Immediately afterwards, the leaves were immersed in a large test tube containing ddH_2_O, sealed in dark condition, and weighed as M2 after 24 h. Then put the blades in the oven to dry until the constant weight is recorded as M3. The relative water content was calculated as:

2 $$\text{r}\text{e}\text{l}\text{a}\text{t}\text{i}\text{v}\text{e}\,\text{w}\text{a}\text{t}\text{e}\text{r}\,\text{c}\text{o}\text{n}\text{t}\text{e}\text{n}\text{t}\,\left(\text{\%}\right)=\frac{M1-M3}{M2-M3}$$

### Chlorophyll *a* Fluorescence Transient

Chlorophyll *a* fluorescence transients were measured on darkadapted (30 min) leaves using a Plant Efficiency Analyser (PEA, Hansatech, U.K.) during 7:00 to ∼9:00 a.m. The JIP test is a conceptual interpretation model that uses the original data from polyphasic fluorescence transients O-J-I-P to calculate specific characteristics of the light phase of photosynthesis. The fluorescence parameter was performed using the PEA Plus software ver. 1.10 (Hansatech Instruments Ltd). Formulae and glossary of terms are presented in Table 3.

**Table 3:** The normalization of chlorophyll *a* fluorescence transient and relative parameters of JIP-test

Terms and Formulae	description
ABS/RC = Mo(1/V_J_) (1/φPo)	absorption flux per reaction center (RC)
TRo/RC = Mo(1/V_J_)	trapped flux per RC
ETo/RC = Mo(1/V_J_) ψEo	electron transport flux per RC
REo/RC = Mo(1/V_J_) ψEo δRo	electron flux reducing end electron acceptors at the Photosystem I (PSI) acceptor side per RC
DIo/RC = ABS/RC -TPo/RC	thermal dissipation energy flux per RC
φPo = TRo/ABS = [1- (*Fo*/*Fm*)]	The maximum quantum yield of primary photochemistr
ψEo = ETo/TRo = (1-V_J_)	The efficiency with which a trapped exaction can move an electron into the electron transport chain further than QA
φEo = ETo/ABS = [1- (*Fo*/*Fm*)] ψEo	the quantum yield of electron transport
φRo = REo/ABS = [1- (*Fo*/*Fm*)] ψEo δRo	the quantum yield of reduction for end electron acceptors of PSI
φDo = DIo/ABS = 1 - φPo = *Fo*/*Fm*	thermal dissipation quantum yield
ABS/CSm = *Fm*	absorption flux per cross section (CS)
TRo/CSm = φPo RC/CSm	trapped flux per CS
ETo/CSm = φEo RC/CSm	electron transport flux per CS
REo/CSm = φRo RC/CSm	electron flux reducing end electron acceptors at the PSI acceptor side per CS
DIo/CSm = ABS/CSm - TRo/CSm	thermal dissipation energy flux per CS

### Gas Exchange, Chlorophyll Fluorescence Measurements and Energy Partitioning Analyses

Leaf gas exchange was measured on the flag leaves with a CIRAS-3 portable photosynthesis system (PP SYSTEMS, CIRAS-3, USA) on a sunny day from 9:00–11:00 a.m. The air temperature is 30 ℃, and the relative humidity of the air is 50%~70%. The net assimilation rate (An), stomatal conductance (*g*_s_), intercellular CO_2_ concentration (Ci), transpiration rate (E) and water use efficiency (WUE = An/ E) were measured at a photosynthetic photon flux density (PPFD) of 1200 µmol•m^− 2^•s^− 1^ and a chamber CO_2_ concentration (*C*a) of 390 µmol•mol^− 1^. The value of An/Ci can approximately reflect the carboxylation efficiency of leaves. About ten flag leaves from WT and the *sdp25* mutant plants were selected to exert gas exchange measurement, respectively.

Modulated chlorophyll fluorescence was measured using an FMS-2 pulse-modulated fluorometer (*Hansatech*, Norfolk, UK). The steady-state fluorescence (*F*s) of the light-adapted leaves was measured using action light, and a 0.8 s saturating light pulse of 8000 µmol•m^− 2^•s^− 1^ was applied to obtain the maximum fluorescence in the light-adapted state (*F*m’). Turning off the action light, and switching on far-red light to preferentially excite PSI to keep the PSII electron transporter in the oxidized state, the minimum fluorescence (*F*o ‘) of light adapted leaves was determined. Then, the leaves were dark-adapted for 20 min prior to determination of the minimum fluorescence (*F*o) by measuring light, while the maximum fluorescence (*F*m) was examined after application of a 0.8 s saturating light pulse of 8000 µmol•m^− 2^•s^− 1^.The following parameters were then calculated (Maxwell and Johnson 2000): the maximum quantum yield of PSII under light-adapted(*F*v’/*F*m’) was defined as (*F*m’ – *F*o’)/*F*m’; effective quantum yield of PSII photochemistry (ΦPSII) was defined as (*F*m’ – *F*s)/*F*m’; photochemical quenching coefficient (*q*_P_) was defined as (*F*m’ – *F*s)/(*F*m’ – *F*o’).The response of electron transport rate to changing light intensity was acquired by decreased PPFD from 2000 to 0 µmol•m^− 2^•s^− 1^. PPFD were in a decreasing series: 2,000, 1,800, 1,600, 1,400, 1,200, 1,000, 800, 600, 400, 200, 100, 50, 25, and 0 µmol•m^− 2^•s^− 1^.

The electron transport rate (ETR) was calculated as:

3 $$\text{E}\text{T}\text{R}={\alpha }\times {\beta }\times {\Phi }\text{P}\text{S}\text{I}\text{I}\times \text{P}\text{P}\text{F}\text{D}$$

where α is the leaf absorbance (α) was assumed as 0.85, β denotes the distribution of electrons between photosynthetic system (PS) I and PSII assumed as 0.5. Then the response curve of photosynthetic electron flow to light is fitted with the mechanism model of photosynthetic electron flow response to light. In other words, the initial slope (*α*), saturated light intensity (PARsat), and maximum photosynthetic electron flow (ETRmax)were obtained.

The quantification of energy partition in PSII was determined by the quantum yield of three de-excitation processes using the chlorophyll’s fluorescence parameters. This analysis is based on the idea that the sum of all the dissipative processes of the energy absorbed by PSII is equal to 1. The fluorescence program was performed and relevant parameters were calculated according to the method described by Quero et al. (2019).

### Electronic Supplementary Material

Below is the link to the electronic supplementary material.

Supplementary Material 1

## Data Availability

All data generated or analyzed during this study are included in this published article and its supplementary information files.

## Abbreviations

An: CO_2_ net assimilation rate
BC: Bulliform cells
CE: Carboxylation efficiency
Chl* a*: Chlorophyll *a*
Chl* b*: Chlorophyll *b*
Ci: Intercellular CO_2_ concentration
Com: Complementation
CP: Cuticular papillae
CRI: CRISPR/Cas9
E: Transpiration rate
EMS: Ethyl methane sulfonate
ETR: Electron transport rate
g_s_: Stomatal conductance
LP: Large papillae
*MLO*: *Mildew Resistance Locus O*
MP: Medium papillae
MV: Minor vein
NPQ: Non-photochemical quenching
OsRac1: Rop/Rac GTPases
OsRopGEF10: Guanine nucleotide exchange factors for Rop
PRONE: Plant-specific Rop nucleotide exchanger
PSI: Photosystem I
PSII: Photosystem II
*q*_P_: Photochemical quenching coefficient
Rop: Rho of plant
SEM: Scanning electron microscopy
SP: Small papillae
TEM: Transmission electron microscopy
WT: Wild-Type
WUE: Water use efficiency
