# Supplementary material for: Small Papillae Regulated by SPD25 are Critical for Balancing Photosynthetic CO2 Assimilation and Water Loss in Rice
Source: Rice (N Y). 2023 Dec 13;16:58. doi: 10.1186/s12284-023-00676-7 (PMC10716080; doi:10.1186/s12284-023-00676-7)

Supplementary Material

Small papillae regulated by *SPD25* are critical for balancing photosynthetic CO_2_ assimilation and water loss in rice

Lin Zhu, Faliang Zeng, Yinpei Liang, Qi Wang, Hongwei Chen, Pulin Feng, Mingqian Fan, Yanshuang Cheng, Jiayu Wang*

Rice Research Institute of Shenyang Agricultural University/Key Laboratory of Rice Biology & Genetic Breeding in Northeast China (Ministry of Agriculture and Rural Areas), Shenyang, 110866, P. R. China

*Corresponding Author: Jiayu Wang, Email: wangjiayu@syau.edu.cn, ricewjy@126.com

**Table S1.** Genetic analysis of the *spd25* mutant

| Cross | Normal plants | small papillae deficient plants | Total | χ^2^(3:1) | *P*-value |
| --- | --- | --- | --- | --- | --- |
| *Spd25*×N91 | 1302 | 422 | 1724 | 0.25 | 0.62 |

**Table S2.** Specific fluxes per active PSII reaction center in flag leaves

| Parameters | WT | *spd25* |
| --- | --- | --- |
| RC/CSm | 682.45±46.23 | 860.4±50.84 ** |
| ABS/RC | 3.73±0.20 | 3.36±0.14 ** |
| TRo/RC | 2.77±0.13 | 2.54±0.09 ** |
| ETo/RC | 1.45±0.08 | 1.37±0.05 * |
| DIo/RC  REo/RC | 0.96±0.09  0.60±0.05 | 0.82±0.09**  0.55±0.05 |

Shown are mean ± SD from three biological replicates each containing ten plants. ^*^ and ^**^ indicate *P* < 0.05 and *P* < 0.01, Student’s *t*-test.

**Table S3.** Energy distribution of the reaction center per unit area in flag leaves

| Parameters | WT | *spd25* |
| --- | --- | --- |
| ABS/CSm | 2540±157.63 | 2884.22±123.83 ** |
| TRo/CSm | 1887.78±137.81 | 2184.44±131.95 ** |
| ETo/CSm | 993±89.71 | 1175.89±85.22 ** |
| DIo/CSm  REo/CSm | 652.22±38.75  410.11±24.30 | 699.78±40.25 *  473.56±40.43 ** |

Shown are mean ± SD from three biological replicates each containing ten plants. ^*^ and ^**^ indicate *P* < 0.05 and *P* < 0.01, Student’s *t*-test.

**Table S4.** Flux ratios of PSII reaction center in flag leaves

| Parameters | WT | *spd25* |
| --- | --- | --- |
| φPo | 0.74±0.01 | 0.75±0.01 |
| ψEo | 0.53±0.01 | 0.54±0.01 |
| φEo | 0.39±0.02 | 0.40±0.02 |
| φD0  φR0 | 0.25±0.01  0.16±0.01 | 0.24±0.01  0.16±0.01 |

Shown are mean ± SD from three biological replicates each containing ten plants. ^*^ and ^**^ indicate *P* < 0.05 and *P* < 0.01, Student’s *t*-test.

**Table S5.** Gas exchange parameters in the WT and the *spd25* mutant

| Parameters | WT | *spd25* |
| --- | --- | --- |
| Ci (μmol•mol^-1^) | 334.33±9.47 | 323.33±10.46* |
| gs (mmol•m^-2^•s^-1^) | 706.68±62.95 | 615.02±56.22* |
| An (μmol•m^-2^•s^-1^) | 23.93±1.56 | 18.27±4.81** |
| E (mmol•m^-2^•s^-1^) | 11.49±0.55 | 9.32±1.44** |
| WUE (μmol•mmol^-1^)  CE (μmol•m^-2^•s^-1^/μmol•mol^-1^) | 2.09±1.61  7.16±0.51 | 1.87±0.23*  5.63±1.38** |

Shown are mean ± SD from three biological replicates each containing ten plants. ^*^ and ^**^ indicate *P* < 0.05 and *P* < 0.01, Student’s *t*-test.

**Table S6.** Primers used in this study

| Primer | Forward Primer Sequence (5' to 3') | Reverse primer Sequence (5' to 3') | Gene description |
| --- | --- | --- | --- |
| RM3575 | CCTGGAATGATGATGGAAGG | GTTTTGCTTCCTGGAAGTGC | Genetic mapping |
| InDel5 | GCAACACCACAACCACAAAC | GTTGCGATCGATAGTTCGGT | Genetic mapping |
| RM5642 | AAAAACCGGCTAATCCCTCC | TTCGATGGGATTGATCGC | Genetic mapping |
| STS3 | GTGCCACTCCGACAAACTGT | TTTCAGAACAGAGGAGGTAG | Genetic mapping |
| STS5 | TGGTACACTTGCATGTTGAG | AATGTGCTTATACGATGG | Genetic mapping |
| InDel7 | ATGGCAACGGAGAAAGTCTG | AGTTGTTGATACCCGGATCG | Genetic mapping |
| InDel8 | TTGGATGGGGTAGTCATCTC | ACATCTTCGATCGATCGCTC | Genetic mapping |
| CRI | GGCAGGTGAGCGAGGCGCTGACGG | AAACCCGTCAGCGCCTCGCTCACC | CRISPR-Cas9 |
| Com | tcctctagagtcgacctgcagCACTCCGACAAACTGTGCAAAA | gccaagcttgcatgcctgcagCGTCAGTGCCATGTCAGATGAA | Complementation |
| *SPD25* | TCGAAATTCTGGAGAGCTTCAG | GTAACGAGTCGAGGTAAGAGTC | *qRT-PCR* |
| Ubiqitin | GCTCCGTGGCGGTATCAT | CGGCAGTTGACAGCCCTAG | *qRT-PCR* |

**Figure S1.** Phenotypes and main agronomic traits of the WT and the *spd25* mutant. (a) Leaf color of 2.5-month-old WT and *spd25* in the paddy field. (b) Heading date, (c) Tiller number, (d) Panicle length, (e) Number of grains per panicle, (f) Seed setting rate, (g) 1000-grain weight and (h) Grain yield per hole of the WT and the *spd25* mutant grown under paddy field conditions (two plants per hole). Shown are mean ± SD from three biological replicates each containing ten plants. * and ** indicate P<0.05 and P<0.01, Student’s t-test.


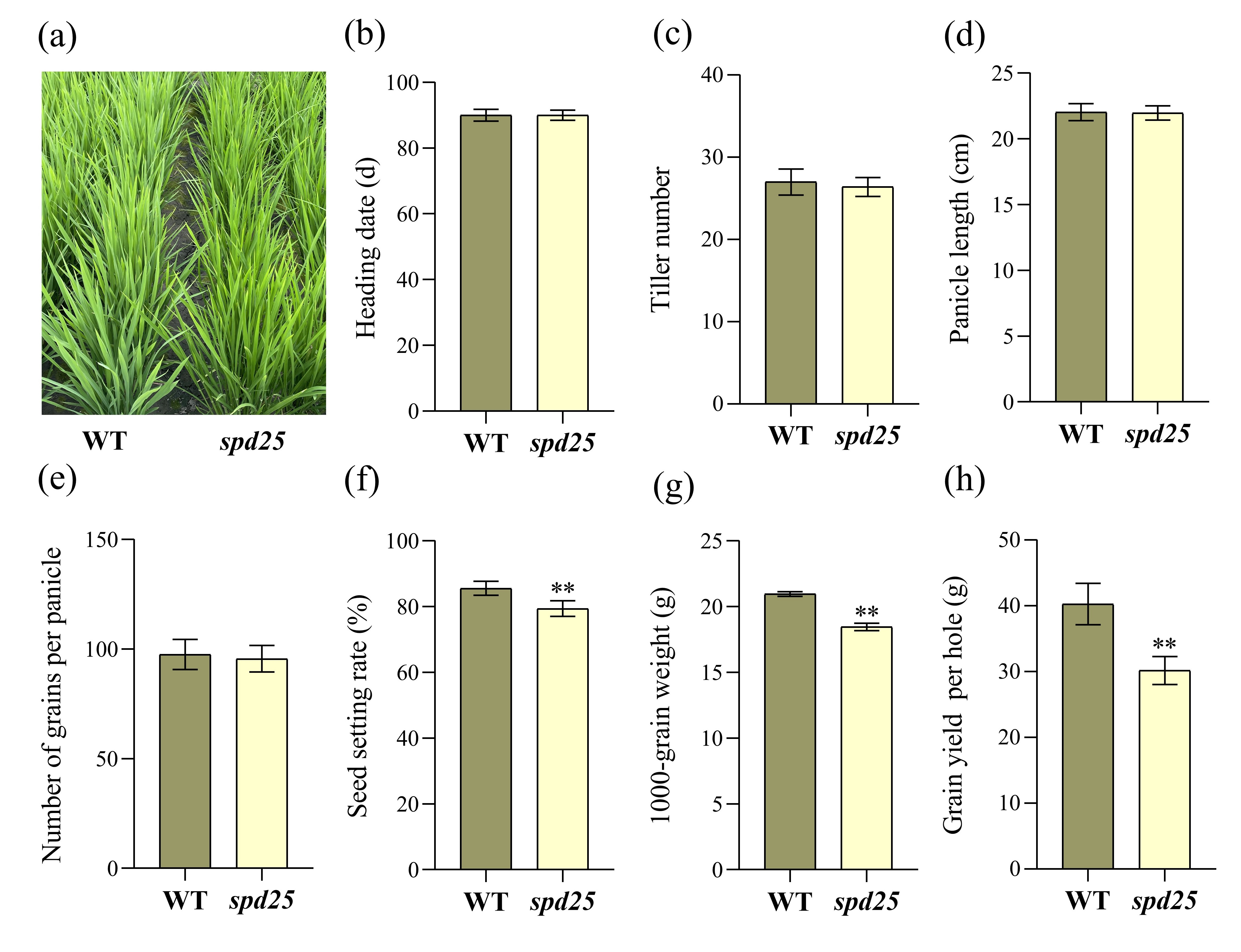


**Figure S2.** CRISPR/Cas9 and complementation analysis of *SPD25* gene. SEM images of the adaxial (a-d) and abaxial (e-h) leaf surfaces in WT, *spd25,* Com and CRI. Com, Complementation; CRI, CRISPR/Cas9. Bars = 90 μm.


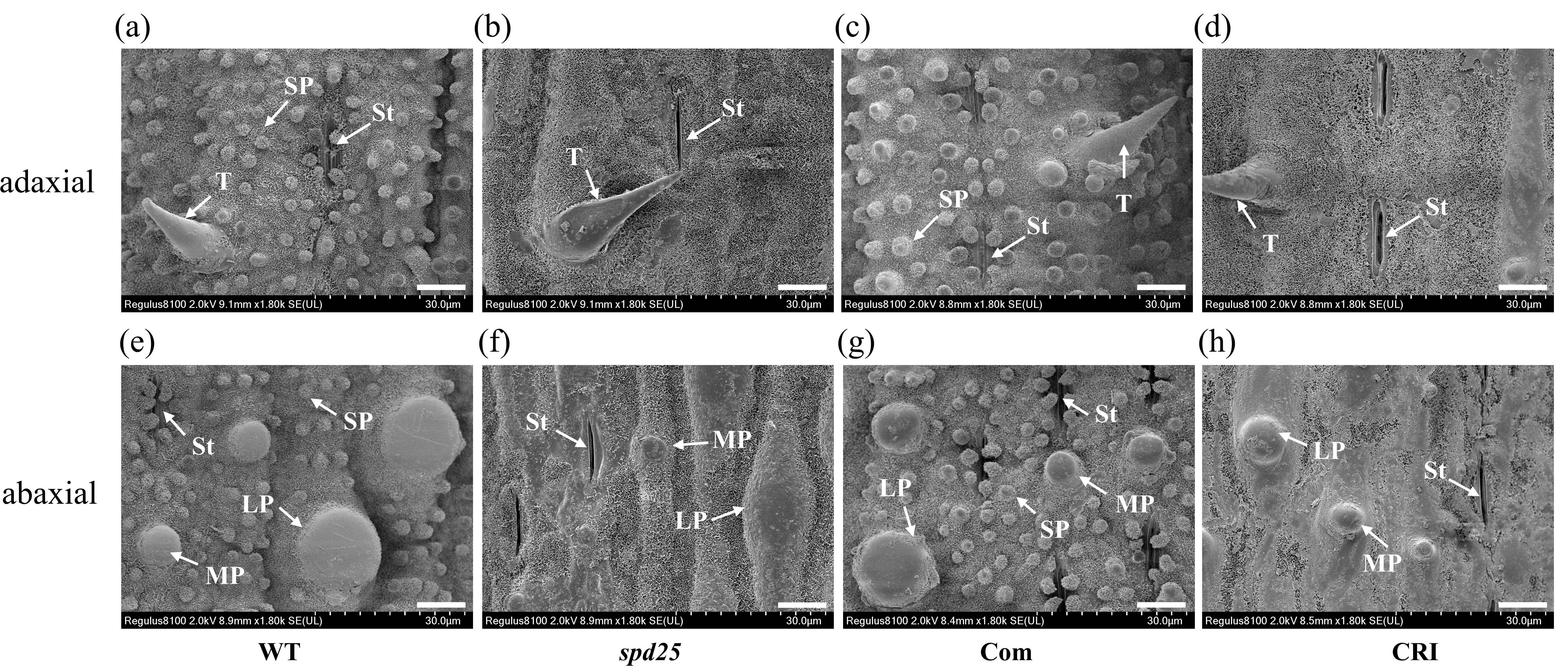


**Figure S3.** Leaf temperature of the WT and the *spd25* mutant. Shown are mean ± SD from three biological replicates each containing ten plants. * and ** indicate P<0.05 and P<0.01, Student’s t-test.


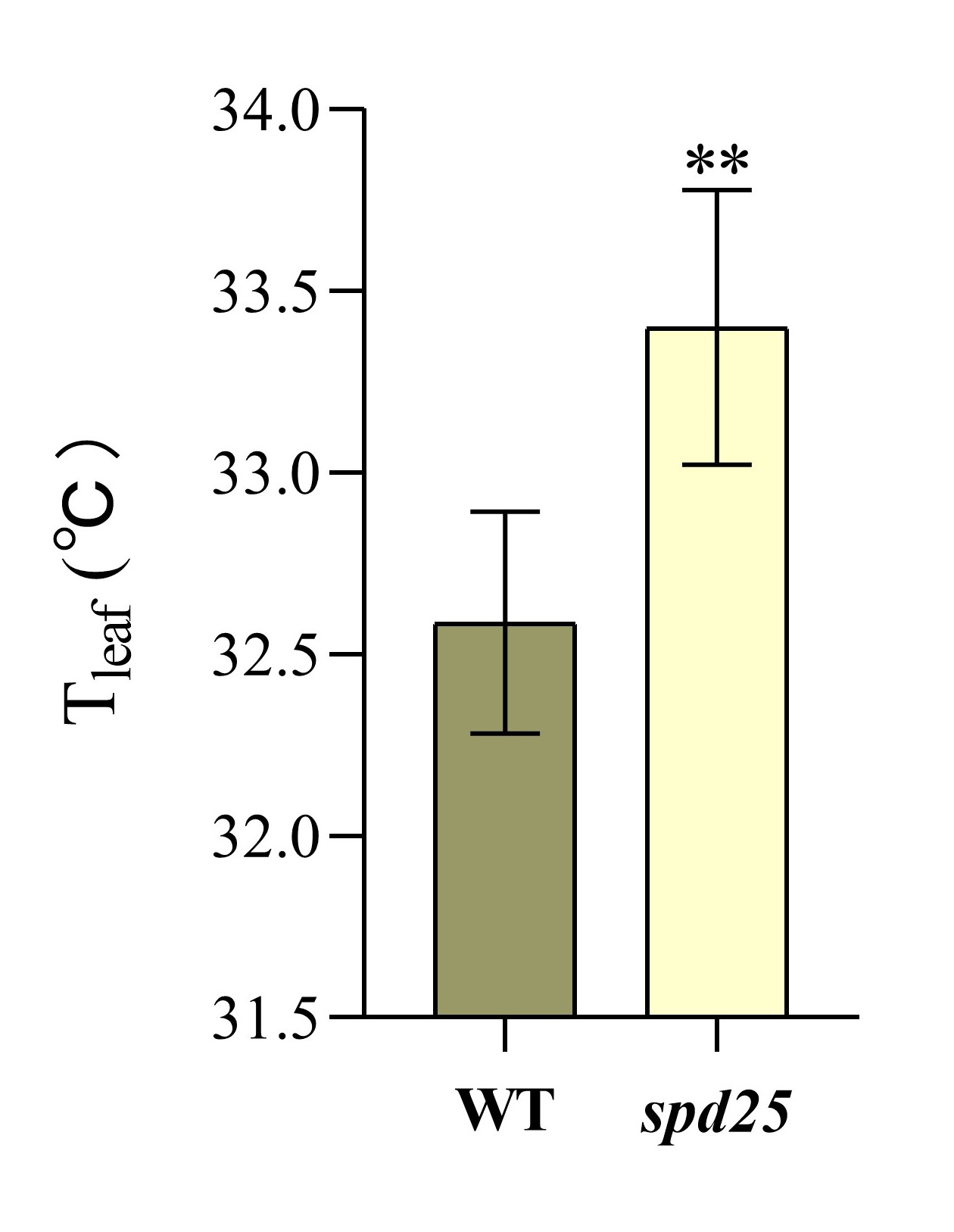

Supplement: Supplementary file 1 — Supplementary Material 1 [file 12284_2023_676_MOESM1_ESM.docx]
